# The architectural design of smart ventilation and drainage systems in termite nests

**DOI:** 10.1126/sciadv.aat8520

**Published:** 2019-03-22

**Authors:** Kamaljit Singh, Bagus P. Muljadi, Ali Q. Raeini, Christian Jost, Veerle Vandeginste, Martin J. Blunt, Guy Theraulaz, Pierre Degond

**Affiliations:** 1 Department of Earth Science and Engineering, Imperial College London, SW7 2AZ London, UK.; 2 Department of Chemical and Environmental Engineering, University of Nottingham, NG7 2RD Nottingham, UK.; 3 Centre de Recherches sur la Cognition Animale (CRCA), Centre de Biologie Intégrative (CBI), Université de Toulouse, CNRS, UPS, Toulouse, France.; 4 School of Chemistry and GeoEnergy Research Centre, University of Nottingham, University Park, NG7 2RD Nottingham, UK.; 5 Department of Mathematics, Imperial College London, SW7 2AZ London, UK.

## Abstract

Termite nests have been widely studied as effective examples for ventilation and thermoregulation. However, the mechanisms by which these properties are controlled by the microstructure of the outer walls remain unclear. Here, we combine multiscale X-ray imaging with three-dimensional flow field simulations to investigate the impact of the architectural design of nest walls on CO_2_ exchange, heat transport and water drainage. We show that termites build outer walls that contain both small and percolating large pores at the microscale. The network of larger microscale pores enhances permeability by one to two orders of magnitude compared to the smaller pores alone, and it increases CO_2_ diffusivity up to eight times. In addition, the pore network offers enhanced thermal insulation and allows quick drainage of rainwater, thereby restoring the ventilation and providing structural stability to the wet nest.

## Teaser

Ventilation and drainage in termite nests are controlled by microscale morphological features of the outer walls.

## INTRODUCTION

Many animals and plants have developed advanced physical structures and operational skills, which have inspired a number of design innovations (*1*–*8*). Among them, termite nests have long been investigated for ventilation and thermoregulation. Their structure maintains a stable temperature throughout the year and permits self-sustainable CO_2_ exchange with the atmosphere for ventilation (*9*–*13*). These self-sustaining temperature and ventilation properties have been a key motivation for designing eco-friendly buildings.

There are two types of termite nest: fungus growing and non–fungus growing. In the former case, termites cultivate fungus for food in the interior fungus-comb chambers of the nest. However, the fungus results in enhanced levels of CO_2_ in the nest (*14*). For the survival of the millions of termites living in the base chambers of the nest, the CO_2_ must be dissipated to the atmosphere. This is mainly achieved by gas exchange through millimeter-scale external openings in the outer wall of the nest. Termites open and close these openings frequently in response to the amount of CO_2_ accumulated in the nest and local breeze outside the nest (*15*). In the outer wall, in addition to the millimeter-scale openings, there are smaller pores that have also been hypothesized as a source of gas exchange for ventilation (*11*, *12*, *16*–*18*). The mechanisms by which termite nests achieve effective ventilation and control the nest temperature have been studied extensively in the past. These mechanisms include temperature-driven convection current ventilation (*11*, *12*, *17*), passive diffusion (*11*), external wind flow (*15*, *19*), metabolic heat (*20*), evaporative cooling (*21*), or a combination of any of these effects.

CO_2_ exchange is more critical in non–fungus-growing closed nests such as *Trinervitermes geminatus* termite nests that have no large millimeter-scale openings in the outer wall and lack specific aeration tunnels (*9*, *22*, *23*). The outer wall contains microscale pores that have been assumed to be disconnected in previous two-dimensional visualization studies (*16*) and is therefore considered to have no external opening to the atmosphere (*10*). It is unclear how these non–fungus-growing termites exchange CO_2_ to the atmosphere for effective ventilation of their nest. In particular, the role of the three-dimensional microscale morphological features of the outer wall in controlling gas exchange and heat transfer is unknown. If the outer wall is porous, then are the pores connected and permeable? And if so, how do they contribute to air circulation or ventilation? In addition, does the porous structure of walls control the thermal insulation and the structural stability of the nest? Resolving these questions will bring us one step closer to understanding mechanisms that will be useful in designing energy efficient buildings (*24*–*26*).

Here, we use multiscale X-ray imaging combined with three-dimensional flow field simulations (*27*), borrowing techniques from research in subsurface flows in rocks (*28*), to provide new insights into the micro- to macroscale architectural design of the outer wall of two non–fungus-growing termite nests from different locations in Africa. Although the materials used for nest construction in the two selected nests are considerably different, we show similarities in the microscale structural features of the outer wall and their role in providing effective CO_2_ exchange to the atmosphere and thermal insulation to the inner parts of the nests. We also demonstrate that these microscale structural features help in rapid water drainage after rain and during nest construction from the moist soil, providing both structural stability and ventilation.

## RESULTS

**Fig. 1:**
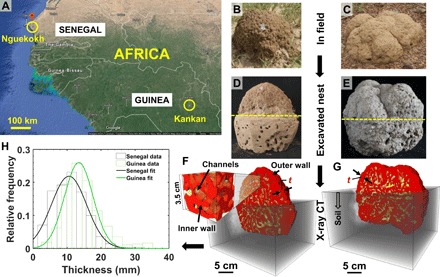
Termite nest locations, excavation, and x-ray tomographic imaging. (**A**) Two sampling locations, Nguekokh (Senegal) and Kankan (Guinea)—marked by yellow circles, were selected for this study. The Senegal nest was taken from a region that is sandy on the surface (brownish in the satellite map), whereas the Guinea sample was taken from a more vegetated region. The map in this image is taken from Google Maps, with the data provider listed at the base of the image (Imagery 2017 Landsat/Copernicus, Data Scripps Institution of Oceanography, National Oceanic and Atmospheric Administration, U.S. Navy, National Geospatial-Intelligence Agency, General Bathymetric Chart of the Oceans, Map data 2017 Google). (**B** and **C**) Nests in the field in Senegal (B) and Guinea (C). (**D** and **E**) These nests were excavated from the field and brought to the laboratory for x-ray imaging. The yellow dashed lines in (D) and (E) show the boundary between the upperground and the underground portions of the nests. [Photo credit for parts (B) to (E): Christian Jost]. (**F** and **G**) The excavated nests were imaged in three dimensions nondestructively with a medical x-ray tomography scanner at a pixel resolution of 0.3 to 0.6 mm. The imaging plane was clipped vertically to show the interiors of both nests. Red and yellow represent solid material and the inner channels of the nest, respectively. (**H**) Histograms of the thickness of the outer walls of the nests, which was measured from the tomographic images (F) and (G) in the upper parts of the nests that were exposed to the atmosphere.

We investigated two African non–fungus-growing *T. geminatus* termite nests (Fig. 1A), one from Nguekokh, Senegal (Fig. 1B) and the other from Kankan, Guinea (Fig. 1C), which are about 1100 km apart. Both nests were constructed by the same termite species. The termites in both nests have a body size of about 3- to 5-mm height and 4- to 14-mm length. We first imaged the excavated nests (Fig. 1, D and E) with a medical x-ray computed tomography (CT) scanner at a voxel resolution of 0.3 to 0.6 mm, which allowed us to investigate the complete structure of the nests in three dimensions nondestructively.

**Fig. 2:**
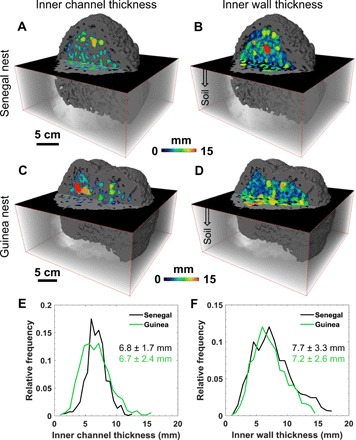
Thickness profiles of the inner solid walls and channels of the nest. (**A** and **B**) Thickness maps of the Senegal nest, showing the width of inner channels (A) and inner solid walls (B). The regions close to the outer wall were not considered in this analysis. (**C** and **D**) Thickness maps of the Guinea nest, showing the width of inner channels (C) and inner solid walls (D). The semitransparent box indicates the parts of the nest that were underground. (**E** and **F**) Histogram of thickness maps of the inner channels (E) and the inner walls (F).

From the medical x-ray CT scanning, both nests appear to have a similar architecture (Fig. 1, F and G). In addition, the thickness of the inner solid walls and channel width is identical (Fig. 2). Here, the channels are defined as millimeter-scale openings in the nest, which are used for passages by termites. The outer walls of the nests (which separate the atmospheric air from the air in inner channels; Fig. 1, F and G) have a thickness of 11.1 ± 4.9 mm (mean ± SD) and 14.9 ± 5.6 mm (Fig. 1H) for the Senegal and Guinea nests, respectively. These values are approximately 1.4 to 2.1 times larger than the values obtained for the inner walls of the nests, which are 7.7 ± 3.3 mm and 7.2 ± 2.6 mm for the Senegal and Guinea nests, respectively (Fig. 2).

From the satellite map (Fig. 1A), it can be seen that the color of the surface soil at the two studied locations differs noticeably (Fig. 1A). The site in Senegal appears sandy (brownish color), whereas the site in Guinea is green with a vegetation cover, indicating a higher clay content and a larger moisture and nutrient retention capacity. This observation is consistent with our x-ray diffraction (XRD) analysis (fig. S1 and table S1), which shows the presence of a majority of quartz minerals with small clay fractions in the Senegal nest and a larger fraction of clay (kaolinite and illite) in the Guinea nest. We hypothesize that the higher clay fraction in the Guinea nest affects the thermal conductivity and other morphological properties of the nest.

### Three-dimensional microstructure of the outer walls of the nests

**Fig. 3:**
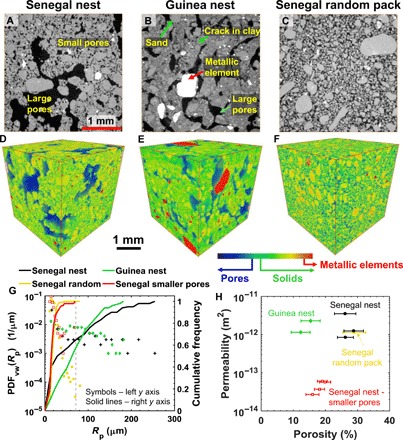
X-ray microtomography analysis of termite nests. (**A** and **B**) Two-dimensional grayscale cross sections of the x-ray microtomographic images of the outer wall of the Senegal (sample 1) (A) and Guinea (sample 1) (B) nests at a voxel size of 5 μm. Gray and white spots represent solid matrix and metallic elements [consistent with the XRD analysis], respectively, while black represents empty (void) space filled with air. In the Senegal nest (A), the smaller pores (intrapellet pore space) and the larger pores (interpellet pore space) are distinguishable, whereas the Guinea nest (B) shows only interpellet pore space, because of a larger fraction of clay indicated by shrinkage cracks in the solid matrix. (**C**) The solid part of the Senegal nest was dissolved in water to form a slurry in a glass vial, which was then air-dried without compaction, hereafter called Senegal random pack, and imaged with x-ray microtomography. (**D** to **F**) Three-dimensional images of the Senegal, Guinea, and Senegal random pack, with their color coding for pores, solids, and metallic elements. (**G**) Probability density function (PDF) and cumulative frequencies of the pore size of the samples shown in (D) to (F). We also show data (red) for a subset (SSc) taken from the Senegal nest sample in which the smaller pores were isolated for pore size comparison (refer to text and fig. S2 for further details). The vertical dashed gray line indicates the upper bound of the pore radii of smaller pores, obtained from subsets SSa-SSd. (**H**) Porosity-permeability relationship for the Senegal, Guinea, Senegal random pack, and four different subsets of isolated smaller pores of the Senegal nest. The error bars of the porosity are extracted from the porosity values of each slice along the *z* axis of the image.

To investigate the microstructural features of the walls of the nest, we drilled multiple samples from the inner and outer walls of the nests (see Materials and Methods) and scanned them nondestructively using a high-resolution x-ray microtomography scanner at voxel sizes of 2 and 5 μm. The notable feature of both nests is the presence of large microscale pores in the walls (Fig. 3, A, B, D, and E). In the Senegal nest (Fig. 3, A and D), a network of large and small pores is observed. The small pores could be interparticle (intrapellet) pore spaces inside individual soil pellets collected by termites. The large pores could be interpellet pore spaces formed during construction when soil pellets were fused together. Here, a soil pellet refers to the soil material collected and shaped by a termite. The volume of pellets has been reported to be of the order of 0.59 ± 0.36 mm^3^ (equivalent to a radius of 0.52 ± 0.44 mm) for *Odontotermes obesus* termites that are similar to *T. geminatus* termites (*29*). If we consider a tetrahedral packing of pellets (considering pellets as spheres), then the radius of inscribed sphere in the pore space between pellets would be approximately 117 μm [=0.225*R*, where *R* is the radius of a pellet (*30*)], which is in the range of pore radii reported in Fig. 3G.

In general, the larger pores (as seen in the walls of the termite nests) are not expected to form if the nest material (e.g., soil particles) was packed randomly as in the case of a random sand pack (*31*, *32*). To test this hypothesis, we prepared a slurry (semiliquid mixture of solid material and water) of the Senegal nest material by dissolving it in water in a glass vial, which was then stirred gently by hand using a thin plastic rod. The slurry was then air-dried without compaction and imaged with x-ray microtomography (Fig. 3, C and F). This air-dried sample will be referred to as the Senegal random pack. The images in Fig. 3 (C and F) show that only interparticle (smaller) pores are present in the Senegal random pack, with pore sizes similar to the smaller pores in the Senegal nest walls (compare the red and yellow lines in Fig. 3G). For this analysis, we isolated the smaller pores in the Senegal nest from the larger pores by taking a cubical subset where the pore space was entirely composed of smaller pores (fig. S2). The average size of these smaller pores in the Senegal nest is 19.3 ± 10.6 μm (mean ± SD), which is of the same order of magnitude as in the Senegal random pack (19.6 ± 6 μm). On the other hand, the average pore size in the complete Senegal nest sample (containing both smaller and larger pores) is 68.5 ± 63.4 μm, which is larger with a wider spread in the pore sizes (Fig. 3G).

Like the Senegal nest sample, the outer wall of the Guinea nest also contains large pores (Fig. 3, B and E). Only a few small pores are observed in the outer walls of the Guinea nest, possibly due to the presence of a larger fraction of clay in the wall material (fig. S1 and table S1). However, we observe various microscale shrinkage cracks in the clayey material (Fig. 3B and fig. S3). The porosity (defined as ratio of the volume of void space in a sample to the total sample volume) of the Guinea nest wall, which is dominated by the contribution of large pores, is 15 ± 2.8% (mean ± SD). Here, we calculated the SD from the porosity values of each two-dimensional slice of an x-ray tomographic image along the axis of the sample. This porosity value is significantly lower than that of the Senegal nest wall 27.9 ± 2.9%. Despite this difference in porosity, the sizes of the pores in the Guinea nest wall (72.9 ± 44.5 μm) are of the same order of magnitude as observed in the Senegal nest sample (Fig. 3G). This similarity in pore sizes occurs due to the dominance of larger pores in both nest walls. In addition, both nest walls show a high percentage of connectivity of the pore space (97 to 98% and 92 to 93% of the pore space in the Senegal and Guinea samples, respectively), which is likely to play a crucial role in air flow and ventilation. For the connectivity analysis, we considered the connected voxels belonging to the pore space, which spanned across the full length of the sample (along an axis).

### Flow and thermal properties—The role of larger microscale interconnected pores

**Fig. 4:**
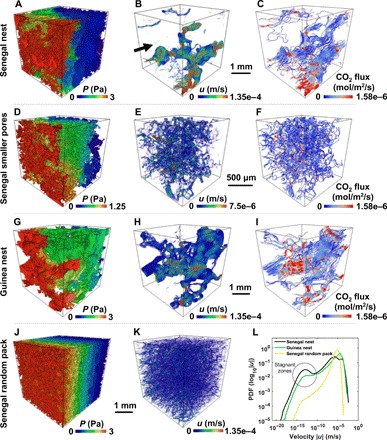
Flow fields and CO_2_ diffusive fluxes. (**A** and **B**) Flow field simulation using Navier-Stokes equation on the Senegal nest (sample 1), showing normalized pressure (A) and velocity (B) fields. The black arrow in (B) shows the inlet face and the direction used in all simulations (A to K). The boundary condition used in the flow simulations corresponds to a pressure gradient of 1 Pa/mm. A higher pressure is imposed on the inlet face of the sample. (**C**) CO_2_ flux visualization in the porous matrix of the Senegal sample, which was estimated by solving Fick’s second law. The boundary condition used in this simulation corresponds to a 5% change in CO_2_ concentration (relative to an atmospheric CO_2_ concentration of ~0.0164 mol/m^3^) across each 1 cm thickness of the nest wall. (**D** to **I**) Similarly, these simulations were conducted on the subsets of the Senegal nest containing smaller pores and the Guinea nest. Pressure (D), velocity (E), and CO_2_ flux (F) in the subset (SSc) containing only smaller pores in the Senegal nest. Pressure (G), velocity (H), and CO_2_ flux (I) in the Guinea nest (sample 1). Pressure (J) and velocity (K) fields in the Senegal random pack. (**L**) PDF of the logarithm of the pore-scale velocities in different samples.

The interconnected larger (microscale) pores in the nest walls are formed either according to simple construction rules similar to the way ant colonies build their nests (*19*, *33*) or as a consequence of physical constraints resulting from the natural way of putting pellets together. These larger pores have many advantages for the nest structure. First of all, the presence of interconnected larger pores results in enhanced permeability. Permeability is the property of a porous medium, which measures the ability of a fluid to flow through it. It is part of the coefficient of proportionality in Darcy’s law that relates pressure drop to fluid velocity (see Eq. 6 in Materials and Methods) and is expressed in the units of square meters (*28*, *34*). The permeability of different samples of the Senegal nests, computed by solving the Navier-Stokes equation (see Materials and Methods), is in the range of 0.6 × 10^−12^ to 3.5 × 10^−12^ m^2^ (Fig. 3H). The computed flow fields are focused in large interconnected pores (Fig. 4, A and B), indicating higher velocities in large pores. To investigate the effect of small and large pores on the permeability and flow fields, we isolated many regions in various tomographic images of the Senegal nest wall by taking cubical subsets at locations where the pore space consisted entirely of smaller pores (fig. S2). The computed permeability of the smaller pores in the isolated subsets is in the range of 2.7 × 10^−14^ to 6 × 10^−14^ m^2^, which is one to two orders of magnitude lower than that in the complete nest sample (Fig. 3H). Qualitatively, the flow fields are uniformly distributed in the smaller pores, with fewer locations where the flow fields are focused with higher flow velocities (Fig. 4, D and E).

We also observe that the flow fields are focused in the larger pores of the outer wall of the Guinea nest sample (Fig. 4, G and H). Although the porosity of the outer wall of the Guinea nest is significantly lower than that of the Senegal nest, the permeability values of 1.1 × 10^−12^ to 2.3 × 10^−12^ m^2^ are in the range of the Senegal samples (Fig. 3H). The permeability of the outer walls of the Guinea nest would be close to zero without larger pores, because the clayey soil with some microscale cracks (fig. S3) is approximately impermeable, providing no air ventilation.

Both the Senegal and Guinea nest samples show a wide distribution of local velocities, with stagnant flow zones at lower velocities predominantly in the Senegal sample (Fig. 4L). The subsets containing smaller pores in the Senegal nest also show stagnant zones (fig. S2), which suggests that the presence of dead-end pores and the clay-blocked throats between neighboring pores could cause this behavior. On the other hand, the Senegal random pack (Fig. 3, C and F), which has comparatively smaller pores (Fig. 3G), has a more uniform flow field (Fig. 4, J and K), without having significant stagnant zones (Fig. 4L). This behavior indicates the presence of a well-connected pore space in the Senegal random pack.

To estimate the apparent CO_2_ diffusivity and CO_2_ flux in the porous matrix of the outer walls of both nests, we solved for transport using Fick’s second law in the pore spaces of these samples (see Materials and Methods and Fig. 4, C, F, and I). The apparent diffusivity of the outer walls of the Senegal nest is in the range of 3.9 × 10^−7^ to 7.2 × 10^−7^ m^2^/s, which is a factor of three to eight larger than that of the smaller pores in the outer wall (8.8 × 10^−8^ to 1.3 × 10^−7^ m^2^/s), indicating that the interconnected larger pores in the Senegal nest enable passive diffusion of the CO_2_. The apparent diffusivity of the outer wall of the Guinea nest (1.9 × 10^−7^ to 3.1 × 10^−7^ m^2^/s) is similar to that of the Senegal nest, indicating that the larger pores have a dominant role in controlling CO_2_ exchange. The CO_2_ flux streamlines also show similarities in the Senegal and Guinea nest samples (Fig. 4, C and I).

The above approximations consider only diffusive CO_2_ exchange. To account for advection due to wind flow outside the nest, we estimate the Péclet number for mass transfer (Pe_m_), which is the ratio of advective-to-diffusive transport flux over the thickness of the outer walls (see Materials and Methods). Considering a wind velocity of 0 to 5 m/s outside the nest and using the values of apparent diffusivity and permeability from numerical simulations, we obtain a Péclet number in the range 0 to 7.35 and 0 to 6.06 for the Senegal and Guinea nest samples, respectively. For higher wind velocities outside the nest, when Pe_m_ > 1, advective transport dominates over diffusion, whereas for Pe_m_ < 1, diffusive exchange of CO_2_ is dominant. The value of Pe_m_ = 1, at which advection and diffusion contribute equally, corresponds to wind velocities of 1.8 to 5 m/s and 2 to 2.3 m/s for samples of the Senegal and Guinea nests, respectively. Overall, these calculations show that both diffusive and advective transports of CO_2_ are important depending on the wind velocity outside the nest.

**Fig. 5:**
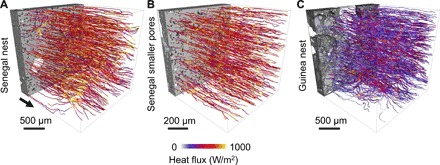
Heat flux simulations. Heat flux streamlines colored by the magnitude of the heat flux obtained for an applied temperature gradient of 1 K/cm in the Senegal nest (**A**), smaller pores in the Senegal nest (SSc) (**B**), and the Guinea nest (**C**). The black arrow in (A) shows the direction of temperature boundary condition used in all simulations (A to C). The light and dark gray in the solid matrix show sand grains and clay, respectively, whereas white represents metallic elements.

We also compute thermal conductivities of each sample by performing steady-state simulations on the heterogeneous solid matrix (see Materials and Methods). The apparent thermal conductivity (here defined as the overall thermal conductivity of a porous medium in the presence of negligible conducting void space) of the outer wall of the Senegal nest, ~4.18 W/m/K, is 16.6% smaller than that computed on a subset containing smaller pores, ~5.01 W/m/K. This difference indicates that the larger pores and higher porosity provide air insulation to the nest. The simulated heat flux streamlines also show small differences (Fig. 5, A and B). On the other hand, because of a higher clay fraction in the walls of the Guinea nest, the apparent thermal conductivity is considerably lower, 1.58 W/m/K, therefore further enhancing thermal insulation by reducing thermal exchange between inner and atmospheric air. The thermal streamlines also show significantly lower heat transfer for the same temperature drop across the Guinea sample (Fig. 5C). The presence of a larger fraction of metallic elements in the Guinea nest (compared to Senegal nest samples) could affect this analysis; however, their poor connectivity and the presence of a larger amount of connected clay minerals could explain the observed behavior.

To compare the advective heat transport through pores with heat conduction through the solid matrix, we calculated the Péclet number for heat transfer (Pe_h_) over the thickness of the outer walls (see Materials and Methods). Using the values of apparent thermal conductivity from numerical simulations, we obtain a Péclet number for heat transfer in the range of 0 to 8.43 × 10^− 4^ and 0 to 1.45 × 10^−3^ for the Senegal and Guinea nest samples, respectively. These low values of the Péclet number indicate that heat transfer is controlled by conduction through the solid material (for wind velocities in the range of 0 to 5 m/s).

From the above analysis, it is clear that the large interconnected pores in both nests are critical for (i) thermal insulation of the inner parts of the nest and (ii) ventilation and CO_2_ exchange. For a higher Péclet number, when the outer wind velocity is high, advective transport dominates over diffusion. In this case, again, the larger pores in the nest wall help the flow of air due to their higher permeability values.

We also note that the porosity of the walls of a fungus-growing nest reported in a previous study (*12*) is considerably larger (37 to 47%) compared to the values observed here. In the future, a similar analysis can be conducted on such high porosity nest walls to compare the pore sizes and flow and thermal properties. Further, for an accurate prediction of effective temperature control of termite nests, it is also important to consider the thermal capacity of the system, particularly the difference between the thermal capacity of the solid matrix and air. However, in this study, our focus is on the effect of small-scale features and how they affect the ventilation, thermal, and drainage properties of the nest walls.

### What happens after rainy periods?

The pores in the nest walls also play a crucial role in the stability of the wet nest during its construction and in ventilation during rainy days. After a short episodic period of rain, the rain water can spread out into the porous matrix. The water from larger pores near the nest surface can be pulled into the smaller pores of the inner parts of the water-wet (hydrophilic) nest material until capillary equilibrium is reached. Under this scenario, the larger interconnected pores become available for gas exchange rapidly, helping in ventilation and further drying of smaller pores that retain water due to capillary forces.

**Fig. 6:**
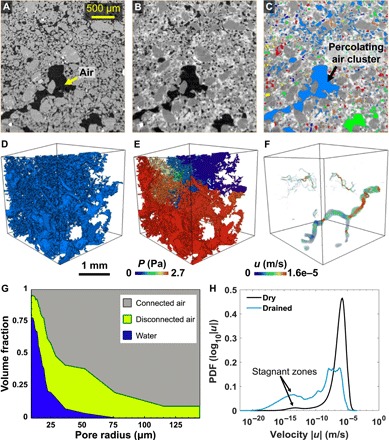
Drainage of the Senegal nest. (**A**) A two-dimensional grayscale cross section of the dry Senegal sample (sample 2). Air is represented by black, and the solid phase is represented by gray. (**B** and **C**) The sample was saturated with 0.16 ml of potassium iodide (KI)–doped water (for improving x-ray absorption contrast, see Materials and Methods) from the top, which was then allowed to drain. The x-ray tomographic image represents the sample at approximately 2.5 hours (B). An axis connectivity analysis was performed on the air phase (C). For the connectivity analysis, the connected voxels belonging to the pore space that spanned across the full length of the sample (along an axis) were considered. Here, light blue represents the connected percolating air cluster spanning across the length of the sample. Various other colors show disconnected air clusters. Light green at the base could be connected; however, because of its presence at the border of the image, it is assigned as a disconnected phase during image processing. (**D**) Three-dimensional visualization of the percolating air cluster in the drained sample. (**E** and **F**) Flow field simulations on the air phase of the drained sample showing pressure (E) and velocity (F) fields. (**G**) Pore occupancy analysis of the drained sample. The plot shows the fraction of the pore space occupied by water [white in (B)], connected air cluster [light blue in (C) and (D)], and disconnected air [various colors in (C)]. (**H**) PDFs of the velocity fields for the dry and the drained Senegal sample. The drained sample shows a considerable amount of stagnant regions with flow focused through fewer open paths.

In the case of prolonged periods of rain, the pores of the nest wall can be filled with water and block the passage of air. To investigate this situation, we conducted a water imbibition experiment on a small sample (sample 2) of the Senegal nest wall. We first imaged the sample under dry conditions, which shows the interconnected larger pores (Fig. 6A) similar to those observed in sample 1 in Fig. 3A. We then injected water from the top of the sample. It was allowed to drain for approximately 2.5 hours and was imaged again. The evaporation during scanning was restricted by wrapping a thin sheet of cling film around the sample. The drained sample is shown in Fig. 6B in which air and water are black and white, respectively. We observe that air is present mostly in larger pores, and the smaller pores remain water-filled. This observation is supported by the pore occupancy analysis shown in Fig. 6G in which the water occupies smaller pore radii, whereas larger pore radii are occupied by connected and disconnected air. Here, the connected air (blue in Fig. 6, C and D) was separated from disconnected air clusters (different colors in Fig. 6C) by performing label analysis and isolating the connected air cluster that spanned across the sample. The disconnected air occupies a range of pore radii with a majority of intermediate sizes (Fig. 6G). The air in these pores can become disconnected because of its entrapment in dead-end pores during water injection (*35*, *36*). It should be noted that some large air-filled pores at the boundary of the image may appear disconnected because of image processing artifacts of boundary voxels (e.g., shown in green in Fig. 6C).

There are two possible reasons for the presence of air in larger pores. First, it is possible that the some air clusters become isolated and trapped during water imbibition, which typically occurs in larger pores for water-wet (hydrophilic) porous materials (*35*). The second possibility is that if the pore space of the sample was completely filled with water, then the water-filled pores could be invaded by percolating air. In this case, air as a nonwetting phase would fill larger pores first due to their lower resistance to air invasion. The higher permeability of the larger pores helps the water to drain at a higher rate. The water in the smaller pores (white in Fig. 6B), which is held because of strong capillary forces, would take many hours to dry (*37*).

The air saturation analysis shows that the 52.9% of the pore space is occupied by air, of which approximately half (51.6% of the air phase) is connected and available for air circulation and ventilation. The permeability through this available space is 2.6 × 10^−14^ m^2^. The flow field simulations show that the majority of flow occurs through a single flow channel (Fig. 6, E and F) and the rest of the available space is stagnant to flow (Fig. 6H). Although the permeability of the drained sample is approximately one order of magnitude smaller than that of the dry sample (8.5 × 10^−13^ m^2^), the percolating air path (connected across the sample) is sufficient to start slow ventilation and drying of the rest of the wet nest wall.

This analysis shows that the presence of larger pores in the outer walls of the nest helps to reestablish ventilation and drying of wet walls after rainy periods. We also performed percolation simulation analysis to quantify the invasion and percolation of air in a completely water-filled sample, which is explained in fig. S7.

### Additional benefits of larger pores during nest construction

During nest construction, termites collect the moist nest material (organomineral) to form pellets. The moist pellets are fused together along with their saliva and fecal matter (also called stercoral mortar) (*22*, *38*). It is important that these moist pellets dry quickly to provide stability to the newly formed wet structure. To achieve this, termites appear to take advantage of the presence of interconnected large pores between different pellets. As shown in the previous section, the air invades the larger pores forming a percolating path. The air invasion creates water-air interfaces in the smaller pore spaces, thereby helping in the evaporation of the rest of the water held by strong capillary forces.

Second, the presence of large pores in the nest walls decreases the solid mass required for nest construction. From material balance, the walls of the Senegal nest have an average porosity of 27.1%, corresponding to a solid material fraction of 72.9%. However, the subset of the nest walls containing only smaller pores has an average porosity of 18.3%, corresponding to a solid material fraction of 81.7%. This implies that the large pores save 10.8% of the solid mass required for nest construction. Similarly, on average, termites save 13.8% of the solid mass required for construction of the walls of the Guinea nest. The use of less material can reduce the overall weight of the nest, therefore lowering the risk of collapse of the moist nest. This hypothesis is valid for the range of porosities observed in the present study. With further increases in porosity, there would be a threshold limit at which the nest would collapse because of the low strength of the scaffold. Further, it is also possible that the use of less material would result in faster nest construction.

## DISCUSSION

Overall, we provide new insights into the three-dimensional microscale morphological features of the walls of two termite nests. The outer walls have an effective network of interconnected microscale small and large pores. Our results suggest that the pore-scale structure of the nest walls is different to what would be produced if the solid grains were deposited at random. Whether these termites create interconnected larger pores according to simple construction rules (*19*, *33*) or as a consequence of physical constraints resulting from the way the pellets are packed remain unclear. However, their construction behavior seems to select structures that provide advantages in terms of effective CO_2_ ventilation and thermal regulation.

Pore-scale flow simulations on x-ray microtomographic images show that the velocity fields are focused in the interconnected network of large microscale pores in the walls of both nests, resulting in a permeability that is one to two orders of magnitude larger than the smaller pores and a CO_2_ diffusivity that is approximately a factor of three to eight larger than that of the smaller pores. The Péclet number analysis shows that both diffusive and advective transports of CO_2_ are important depending on the wind velocity outside the nest. Furthermore, the presence of larger pores and the resultant higher porosity in the nest walls also help to reduce the thermal conductivity and temperature exchange between the atmospheric and internal air, therefore providing enhanced thermal insulation to the inner parts of the nest. The presence of a larger percentage of clay in the Guinea nest samples further increases the thermal insulation of the nest.

The drainage experiment and air percolation analysis indicate that the larger pores in the walls allow rapid drainage of the water. This is beneficial for drying of the nest walls during construction, providing structural stability to the moist pellets. Similarly, after rain, the water drains through the interconnected larger pores of the nest walls, allowing the reestablishment of CO_2_ ventilation and providing structural stability to the nest. The presence of larger pores can reduce the solid material required for nest construction by ~11 to 14% compared to the case when the wall material solely consists of smaller pores. This is energetically favorable and decreases the overall weight of the nest, which can reduce the risk of nest collapse during construction. It is also possible that the use of less material can speed up nest construction.

The above findings present a major advancement in our understanding of various processes that control ventilation, thermal regulation, and drainage of termite nests at much smaller scales than previously considered. Future work could focus on similar analyses of the porous walls of fungus-growing termite nests for which air flow, CO_2_, and temperature data are available, for example, from India and Namibia (*11*, *17*). This will then allow unifying the knowledge obtained from different types of nests for a complete understanding of microscale features and their influence on large-scale processes.

## MATERIALS AND METHODS

### Sample collection

Two *T. geminatus* termite nests were excavated in Africa. The first nest was collected from Nguekokh, Senegal, which appears to be constructed on a sandy area (Fig. 1A). The second nest was collected from a green area in Kankan, Guinea. These two site locations are approximately 1100 km apart and differ in soil and vegetation and climate conditions. Both nests were constructed by the same species of termites, the length and height of which are in the range of 4 to 14 mm and 3 to 5 mm, respectively. Both nests were in the Savanna-type vegetation, with higher tree density in Guinea. Both nests were exposed to the sun. The Guinea sample was collected early July 2007, and the Senegal sample was collected early October 2010.

### Medical x-ray CT imaging and image analysis

The excavated nests were scanned with a medical x-ray CT scanner (Somatom Sensation16, Siemens, Erlangen, Germany) with 120-kV and 150-mA settings and with a voxel size of 0.55 × 0.55 × 0.6 mm and 0.58 × 0.58 × 0.3 mm for the Senegal and Guinea nests, respectively. The tomographic images were filtered with a nonlocal means edge-preserving filter, followed by their segmentation into two phases (solid and air) with a seeded watershed algorithm based on the grayscale gradient and grayscale intensity of each voxel using Avizo-9 software. The segmented image was used for three-dimensional visualization using Avizo-9 software. To estimate the thickness of inner solid walls and channels, the solid phase was first isolated. A sequential dilation of 12 pixels and an erosion of 32 pixels were applied on the solid phase of the Senegal nest, whereas a sequential dilation of 20 pixels and an erosion of 50 pixels were applied on the solid phase of the Guinea nest. The resultant image was applied as a mask on all the channels, which isolated the internal channels by removing the channels near the outer wall (Fig. 2). The reason for using a sequential dilation and erosion mask was to analyze the thickness profiles away from the outer walls and to focus on the internal features. We then estimated the width of these isolated inner channels using ImageJ software, which is based on the Euclidean distance and medial axis. Similarly, we estimated the thickness of the inner solids (Fig. 2). The thickness of the outer wall was measured manually from the tomographic images at more than 300 points across the entire outer wall of the nests.

### Sample preparation for x-ray microtomography, imaging and image analysis

We selected various parts of the outer wall of each nest for x-ray microtomographic imaging. In addition, for the Senegal nest, we also scanned the inner wall of the nest (sample 3), which showed similar pore morphologies as for the outer walls. The selected parts were drilled into a cylinder with a diameter of 7 to 12 mm and a height of 10 to 22 mm. These samples were then mounted on a sample holder, which was tailor-made to fit on the rotation stage of the scanner. Each sample was imaged with a voxel size of 2 and 5 μm using a Zeiss Xradia 500 Versa 3D x-ray microscope with 80- to 100-kV and 7- to 9-W settings. Depending on the diameter of the sample, a total of 2400 to 4000 projections were acquired for a 5-μm voxel size dataset, whereas 4000 to 6000 projections were acquired for a 2-μm voxel size dataset. The projection data were reconstructed using proprietary software provided by Zeiss and processed using Avizo-9 software, unless otherwise specified. The tomograms were preprocessed to remove distorted outer regions and filtered with a nonlocal means edge-preserving filter to increase the signal-to-noise ratio. The tomograms were then segmented into two phases, i.e., solid and air (or pore space), with a seeded watershed algorithm based on the grayscale gradient and grayscale intensity of each voxel. The pore radius was estimated from the radius of maximal inscribed spheres located on a medial axis transformation of the pore space (*39*).

The total number of voxels of each phase (air and solid) was computed from the segmented image. The porosity was then calculated using ϕ = pore volume/(pore volume + solid volume). To investigate the effect of the size of the image on the porosity, we conducted a representative elementary volume (REV) analysis. For this, various cubical subsets with increasing side length were taken, from which the porosity was calculated. Figure S4 shows a plot of normalized porosity against the side length of the cubical volume of the image. Here, the porosity is normalized to its final value at 3-mm side length. It is clear from these plots that the REV is achieved at approximately 2.2-mm side length of a cubical volume, giving us confidence in the measurement of overall porosities of the samples from the tomographic images.

The Guinea sample was segmented into four phases, i.e., air, clay, quartz, and hematite (fig. S5), which was then used in simulations for thermal conductivity. The gray scale between clay and quartz was in a narrow range, therefore making the segmentation process difficult. For an effective segmentation of these phases, we used a watershed algorithm based on the grayscale gradient and grayscale intensity of each voxel with an additional top-hat transform on various phases. The top-hat transform (filtering) detects the dark or light intensity areas, corresponding to valleys or the narrow peaks in the grayscale histogram, which is useful in segmenting small features and regions that have low x-ray absorption contrast. Similarly, the Senegal samples were segmented into three phases, i.e., air, quartz, and metallic elements.

### Flow simulations for permeability, CO_2_ diffusivity, and apparent thermal conductivity

For flow simulations, we used an open source computational fluid dynamics (CFD) software, OpenFOAM (*27*). First, the pore space was isolated from the segmented images. The simulations were performed on the pore space images directly by converting each pore voxel of the segmented image into a computational grid cell of the same size. The pressure and velocity fields were then obtained by solving the mass momentum equations using a second-order cell-centered finite-volume method

$$\rho (\frac{\partial u}{\partial t}+u.\nabla u)=-\nabla p+\mu \nabla^{2}u$$ (1)

∇.u=0 (2)

where *u* is the velocity vector and *p* is the pressure. The inlet face and the direction of flow used in all simulations are marked by the black arrow in Fig. 4B. The simulations were performed by applying a fixed relative pressure of 1 Pa at one side of the domain and 0 Pa at the opposite side (outlet). On all the other sides and on the solid walls, we applied a no-slip boundary condition. Since the effect of inertial forces is negligible here, the results can be scaled to correspond to other pressure drops as long as the Reynolds number is sufficiently low, which is in the range of 1.88 × 10^−7^ to 2.15 × 10^−6^ in all our simulations. The simulations were run until a steady-state solution of the equation (∂*u*/∂*t* = 0) was achieved. These computations result in pressure and velocity fields as well as the permeability of the samples. This approach has been validated and used in previous works (*27*, *40*).

The molecular diffusivity of CO_2_ through a porous matrix of the nest samples was simulated by solving Fick’s second law

$$\frac{\partial c}{\partial t}-D_{c}.\nabla^{2}c=0$$ (3)

where *c* is the concentration of the CO_2_ (mol/m3) and *D*_c_ is the diffusion coefficient of CO_2_ in air [≈1.6 × 10^−5^ m^2^/s (*41*)]. Here, the diffusion coefficient of the solid phase is zero, and we assumed that there is no reaction occurring at the fluid-solid interface. The simulations were run by applying a fixed relative concentration of 1 at one side of the domain and 0 at the opposite side (outlet). On all the other sides and on the solid walls, we applied a no-flow (zero-gradient) boundary condition. The results are scaled so that this corresponds to a 5% change in CO_2_ concentration (*12*) (relative to an atmospheric CO_2_ concentration of 0.0164 mol/m^3^) across each 1 cm of the nest wall.

In general, the heat conduction problem in a homogeneous material is solved by Fourier’s law. However, in a heterogeneous material, the heat conduction is more complicated. Under steady-state conditions, the heat conduction can be described as

$$\nabla .(\kappa_{\beta }\nabla T)=0$$ (4)

where κ_β_ is the thermal conductivity (W/m/K) of the β phase and *T* is the temperature field (K). This allowed us to solve Eq. 4 for different phases in a three-dimensional segmented image by assigning different values of thermal conductivity to each phase: 0.96 W/m/K for dry kaolinite clay (*42*), 7.8 W/m/K for quartz (*43*), and 6.4 W/m/K for hematite (*44*). Air was considered as a thermal insulator. The simulations were performed by applying a fixed constant temperature at one side of the domain and a relative temperature of 0 at the opposite side (outlet). On all the other sides and on the solid walls, we applied a no-flow (zero-gradient) boundary condition. The inlet face and the direction of temperature flow used in all simulations are marked by the black arrow in Fig. 5A. These simulations produced temperature fields and the apparent thermal conductivity of the solid matrix. The results were scaled so that this corresponds to a temperature gradient of 1 K/cm across the outer walls of the nest.

### Dimensionless numbers

To account for air advection through microscale pores due to wind flow outside the nest, we estimated the Péclet number. The Péclet number for mass transfer (Pe_m_), the ratio of advective-to-diffusive transport flux over the thickness of the outer walls, is defined as

$${\text{Pe}}_{m}=\frac{U_{D}L}{D}$$ (5)

where *L* (meters) is the characteristic length considered as the average thickness of the nest wall (11.1 and 14.9 mm for the Senegal and Guinea nests, respectively) and *D* (m^2^/s) is the apparent diffusivity of CO_2_ through porous walls of the nest (estimated from numerical simulations described in the previous section). The apparent or Darcy velocity (*U*_D_) of air through the nest wall can be calculated from Darcy’s law

$$U_{D}=\frac{k\Delta P}{\mu \Delta x}$$ (6)

where *k* is the permeability of the nest, which is estimated from direct numerical simulations, and Δ*x* is the thickness of nest wall. Considering a wind velocity of 0 to 5 m/s outside the nest, we obtained a pressure drop (due to wind flow) of 0 to 15 Pa using Δ*P* = ρ*v*^2^/2, where ρ is the density of air and *v* is wind velocity (*12*).

To compare the advective heat transport through pores with heat conduction through solid matrix, we calculated the Péclet number for heat transfer (Pe_h_) over the thickness of the outer walls

$${\text{Pe}}_{h}=\frac{U_{D}L}{\alpha }$$ (7)

where α is the thermal diffusivity, which is calculated as

$$\alpha =\frac{K}{\rho c_{p}}$$ (8)

where *K* is the apparent thermal conductivity (W/m/K) of the solid material of the outer wall of the nest (estimated from numerical simulations described in the previous section) and *c*_p_ is the specific heat capacity of air (J/kg/K).

### Percolation threshold analysis

For computing a percolation threshold, the pore space was isolated from the segmented image (fig. S6A). A local thickness map was computed from the pore space voxels (using ImageJ software). For this computation, the algorithm inserts spheres (using a distance map in the pore space), with a radius that can fit into the pore space along the medial axis (fig. S6B). An interactive thresholding (using Avizo-9 software) was then applied to find the minimum (threshold) radius of the sphere at which the pore space is connected across the image axes. Figure S8C shows the image of the spheres (converted to voxel data) that have a minimum (threshold) radius of 17 μm at which the pore space is connected across the vertical axis. Different colors represent disconnected clusters of the pore space at this particular threshold. The connected cluster is then isolated using an axis connectivity algorithm (fig. S6D). The connected cluster from fig. S6D is masked on fig. S6B to obtain the radius distribution of the connected and percolating pore space (fig. S6E). Figure S6F shows the image with a threshold radius of 17.5 μm at which the pore space is not connected across any axis. The disconnection is marked by a dashed (white) circle. The image processing protocol in fig. S6 shows an example for computing a percolation threshold radius in two-dimensional pore space. In three dimensions, a higher threshold radius is expected as we show in Results.

### Drainage experiment

Sample 2 of the Senegal nest wall, which was prepared for x-ray microtomography imaging, was mounted on a sample holder. A total volume of 0.16 ml of 23 weight % potassium iodide (KI) (puriss, 99.5%, Sigma-Aldrich, UK) aqueous solution was injected at the top of the sample. The water was doped with KI to enhance the x-ray contrast between air and the aqueous phase (Fig. 6). The sample was then wrapped with a cling film to avoid any evaporation of the aqueous phase during scanning. The tomographic image represents the sample at approximately 2.5 hours. The experiment was conducted at ambient temperature (20°C).

The air phase was segmented with a seeded watershed algorithm based on the grayscale gradient and grayscale intensity of each voxel. An axis connectivity analysis was performed on the isolated air phase using Avizo-9 software to confirm the percolation of the air phase between the two sides of the sample.

### XRD analysis

The XRD analyses were performed on both bulk powder samples and clay fraction separated from the sample. The nest material was crushed using mortar and pestle to obtain a fine powder. The powder samples were analyzed using an PANalytical X-Pert Pro, using Cu K_α_ radiation at 40 kV and 40 mA. The samples were scanned over a sampling range of 2.5 to 70°2θ with a step size of 0.0066 and a scan speed of 0.023°2θ per second. For each nest, triplicate samples were used for bulk analyses and duplicate samples for separated clay fraction analyses with and without chemical treatment. As the clay particles (<2 μm in size) generally do not show clear diffraction patterns in bulk samples with random orientation, the clay fraction was separated from the bulk sample upon dispersion of the powder using an ultrasonic bath. Centrifugation of the samples at 1000 rpm for 2 min was used to separate the heavier fraction, whereas the clay was left in suspension. The clay suspended fluid was then put on glass rounds, the clay was allowed to settle, and the material was left to dry to generate oriented mounts for XRD analysis. The air-dried clay fraction samples and glycol-solvated clay fraction samples were used to identify different clays present in the samples. Moreover, some clay fractions were treated with 1 N HCl to confirm the absence of chlorite in the samples.

The diffraction analyses on the bulk samples of both nests show peaks of quartz (*45*); however, the Guinea sample also shows the presence of kaolinite and hematite (fig. S1). The XRD analysis on the separated clay fraction shows peaks of kaolinite and smectite in the Senegal sample and peaks of kaolinite and illite in the Guinea samples. The integrated intensities of the characteristic peaks were used for quantification of the relative mineral abundance in the samples. Separated fractions of different minerals are reported in table S1.

## Supplementary Material

http://advances.sciencemag.org/cgi/content/full/5/3/eaat8520/DC1

Download PDF

## SUPPLEMENTARY MATERIALS

Supplementary material for this article is available at http://advances.sciencemag.org/cgi/content/full/5/3/eaat8520/DC1 Air Percolation Analysis Fig. S1. XRD analysis of the nest material. Fig. S2. Subset selection for the smaller pores in the Senegal nest. Fig. S3. High-resolution x-ray microtomographic images. Fig. S4. REV analysis. Fig. S5. Four-phase separation of the Guinea nest sample. Fig. S6. Computation of percolation threshold. Fig. S7. Air percolation analysis in the outer wall of the termite nests. Table S1. Mineral compositions of the Senegal and Guinea nest material obtained from the XRD analysis. Reference (*46*)

## References

[1] Perna A., Theraulaz G., When social behaviour is moulded in clay: On growth and form of social insect nests. J. Exp. Biol. 220, 83–91 (2017). 28057831 10.1242/jeb.143347

[2] Bar-Cohen Y., Biomimetics—Using nature to inspire human innovation. Bioinspir. Biomim. 1, P1–P12 (2006). 17671297 10.1088/1748-3182/1/1/P01

[3] Autumn K., Sitti M., Liang Y. A., Peattie A. M., Hansen W. R., Sponberg S., Kenny T. W., Fearing R., Israelachvili J. N., Full R. J., Evidence for van der Waals adhesion in gecko setae. Proc. Natl. Acad. Sci. U.S.A. 99, 12252–12256 (2002). 12198184 10.1073/pnas.192252799 PMC129431

[4] Hinman M. B., Jones J. A., Lewis R. V., Synthetic spider silk: A modular fiber. Trends Biotechnol. 18, 374–379 (2000). 10942961 10.1016/s0167-7799(00)01481-5

[5] Bonabeau E., Dorigo M., Theraulaz G., Inspiration for optimization from social insect behaviour. Nature 406, 39–42 (2000). 10894532 10.1038/35017500

[6] E. Bonabeau, M. Dorigo, G. Theraulaz, *Swarm Intelligence*: *From Natural to Artificial Systems* (Oxford Univ. Press, 1999).

[7] P. N. Bartlett, J. W. Gardner, *Electronic Noses: Principles and Applications* 264 (Oxford Univ. Press, 1999).

[8] J. S. Turner, R. C. Soar, Beyond biomimicry: What termites can tell us about realizing the living building, in *First International Conference on Industrialized, Intelligent Construction* (I3CON, 2008), pp. 215–231.

[9] J. Korb, Termite mound architecture, from function to construction, in *Biology of Termites: A Modern Synthesis*, D. E. Bignell, Y. Roisin, N. Lo, Eds. (Springer, 2011), pp. 349–373.

[10] Field M. A., Duncan F. D., Does thermoregulation occur in the mounds of the harvester termite, *Trinervitermes trinervoides* (Sjöstedt) (Isoptera: Termitidae)? Afr. Entomol. 21, 45–57 (2013).

[11] Korb J., Linsenmair K. E., Ventilation of termite mounds: New results require a new model. Behav. Ecol. 11, 486–494 (2000).

[12] King H., Ocko S., Mahadevan L., Termite mounds harness diurnal temperature oscillations for ventilation. Proc. Natl. Acad. Sci. U.S.A. 112, 11589–11593 (2015). 26316023 10.1073/pnas.1423242112 PMC4577200

[13] Korb J., Thermoregulation and ventilation of termite mounds. Naturwissenschaften 90, 212–219 (2003). 12743703 10.1007/s00114-002-0401-4

[14] Konaté S., Roux X. L., Verdier B., Lepage M., Effect of underground fungus-growing termites on carbon dioxide emission at the point- and landscape-scales in an African savanna. Funct. Ecol. 17, 305–314 (2003).

[15] Turner J. S., On the mound of *macrotermes michaelseni* as an organ of respiratory gas exchange. Physiol. Biochem. Zool. 74, 798–822 (2001). 11731972 10.1086/323990

[16] Cosarinsky M. I., The nest growth of the neotropical mound-building termite, *Cornitermes cumulans*: A micromorphological analysis. J. Insect Sci. 11, 122 (2011). 22224433 10.1673/031.011.12201 PMC3281366

[17] Ocko S. A., King H., Andreen D., Bardunias P., Turner J. S., Soar R., Mahadevan L., Solar-powered ventilation of African termite mounds. J. Exp. Biol. 220, 3260–3269 (2017). 28931718 10.1242/jeb.160895

[18] Josens G., Variations thermiques dans les nids de *Trinervitermes geminatus* Wasmann, en relation avec le milieu extérieur dans la savane de Lamto (Côte d’Ivoire). Insectes Soc. 18, 1–13 (1971).

[19] Turner J. S., Termites as models of swarm cognition. Swarm Intell. 5, 19–43 (2011).

[20] Lüscher M., Air-conditioned termite nests. Sci. Am. 205, 138–147 (1961).

[21] Jones J. C., Oldroyd B. P., Nest thermoregulation in social insects. Adv. Insect Physiol. 33, 153–191 (2006).

[22] P. P. Grassé, *Termitologia, vol. II* Fondation des Sociétés – Construction (Masson, Paris, 1984).

[23] C. Noirot, J. P. E. C. Darlington, Termite nests: Architecture, regulation and defence, in *Termites: Evolution, Sociality, Symbioses, Ecology*, T. Abe, D. E. Bignell, M. Higashi, Eds. (Springer, 2000), pp. 121–139.

[24] John G., Clements-Croome D., Jeronimidis G., Sustainable building solutions: A review of lessons from the natural world. Build. Environ. 40, 319–328 (2005).

[25] Ball P., For sustainable architecture, think bug. New Sci. 2748, 35–37 (2010).

[26] A. Khan, *Adapt: How Humans are Tapping into Nature’s Secrets to Design and Build a Better Future* (St. Martin’s Press, 2017).

[27] Muljadi B. P., Blunt M. J., Raeini A. Q., Bijeljic B., The impact of porous media heterogeneity on non-Darcy flow behaviour from pore-scale simulation. Adv. Water Resour. 95, 329–340 (2016).

[28] M. J. Blunt, *Multiphase Flow In Permeable Media: A Pore-Scale Perspective* (Cambridge Univ. Press, 2017).

[29] Zachariah N., Das A., Murthy T. G., Borges R. M., Building mud castles: A perspective from brick-laying termites. Sci. Rep. 7, 4692 (2017). 28680035 10.1038/s41598-017-04295-3 PMC5498601

[30] Avery R. G., Ramsay J. D. F., The sorption of nitrogen in porous compacts of silica and zirconia powders. J. Colloid Interface Sci. 42, 597–606 (1973).

[31] Scheel M., Seemann R., Brinkmann M., Di Michiel M., Sheppard A., Breidenbach B., Herminghaus S., Morphological clues to wet granular pile stability. Nat. Mater. 7, 189–193 (2008). 18264104 10.1038/nmat2117

[32] Mukunoki T., Miyata Y., Mikami K., Shiota E., X-ray CT analysis of pore structure in sand. Solid Earth 7, 929–942 (2016).

[33] Khuong A., Gautrais J., Perna A., Sbaï C., Combe M., Kuntz P., Jost C., Theraulaz G., Stigmergic construction and topochemical information shape ant nest architecture. Proc. Natl. Acad. Sci. U.S.A. 113, 1303–1308 (2016). 26787857 10.1073/pnas.1509829113 PMC4747701

[34] B. J. *Dynamics of Fluids in Porous Media* (American Elsevier Publishing Company Inc., 1972).

[35] Singh K., Menke H., Andrew M., Lin Q., Rau C., Blunt M. J., Bijeljic B., Dynamics of snap-off and pore-filling events during two-phase fluid flow in permeable media. Sci. Rep. 7, 5192 (2017). 28701699 10.1038/s41598-017-05204-4 PMC5507864

[36] Singh K., Scholl H., Brinkmann M., Di Michiel M., Scheel M., Herminghaus S., Seemann R., The role of local instabilities in fluid invasion into permeable media. Sci. Rep. 7, 444 (2017). 28348395 10.1038/s41598-017-00191-y PMC5427855

[37] Shahidzadeh-Bonn N., Azouni A., Coussot P., Effect of wetting properties on the kinetics of drying of porous media. J. Phys. Condens. Matter 19, 112101 (2007).

[38] Brossard M., López-Hernández D., Lepage M., Leprun J.-C., Nutrient storage in soils and nests of mound-building *Trinervitermes* termites in Central Burkina Faso: Consequences for soil fertility. Biol. Fert. Soils 43, 437–447 (2007).

[39] Raeini A. Q., Bijeljic B., Blunt M. J., Generalized network modeling: Network extraction as a coarse-scale discretization of the void space of porous media. Phys. Rev. E 96, 013312 (2017). 29347276 10.1103/PhysRevE.96.013312

[40] Mostaghimi P., Blunt M. J., Bijeljic B., Computations of absolute permeability on micro-CT images. Math. Geosci. 45, 103–125 (2013).

[41] He M., Guo Y., Zhong Q., Zhang Y., Determination of binary gas diffusion coefficients using digital holographic interferometry. J. Chem. Eng. Data 55, 3318–3321 (2010).

[42] Park K., Lee J., Yoon H.-K., Kim D., Hydraulic and thermal conductivities of kaolin–silica mixtures under different consolidation stresses. Mar. Georesour. Geotec. 34, 532–541 (2016).

[43] Fjeldskaar W., Christie O. H. J., Midttømme K., Virnovsky G., Jensen N. B., Lohne A., Eide G. I., Balling N., On the determination of thermal conductivity of sedimentary rocks and the significance for basin temperature history. Petrol. Geosci. 15, 367–380 (2009).

[44] Mølgaard J., Smeltzer W. W., Thermal conductivity of magnetite and hematite. J. Appl. Phys. 42, 3644–3647 (1971).

[45] d’Amour H., Denner W., Schulz H., Structure determination of α-quartz up to 68 × 10^8^ Pa. Acta Cryst. B 35, 550–555 (1979).

[46] Vargaftik N. B., Volkov B. N., Voljak L. D., International tables of the surface tension of water. J. Phys. Chem. Ref. Data 12, 817–820 (1983).

