# Supplementary material for: The architectural design of smart ventilation and drainage systems in termite nests
Source: Sci Adv. 2019 Mar 22;5(3):eaat8520. doi: 10.1126/sciadv.aat8520 (PMC6430624; doi:10.1126/sciadv.aat8520)
Supplement: http://advances.sciencemag.org/cgi/content/full/5/3/eaat8520/DC1 [file supp_5_3_eaat8520__index.html]

Science Advances | Science Advances

## Supplementary Materials

**This PDF file includes:**

- Air Percolation Analysis
- Fig. S1. XRD analysis of the nest material.
- Fig. S2. Subset selection for the smaller pores in the Senegal nest.
- Fig. S3. High-resolution x-ray microtomographic images.
- Fig. S4. REV analysis.
- Fig. S5. Four-phase separation of the Guinea nest sample.
- Fig. S6. Computation of percolation threshold.
- Fig. S7. Air percolation analysis in the outer wall of the termite nests.
- Table S1. Mineral compositions of the Senegal and Guinea nest material obtained from the XRD analysis.
- Reference (*46*)

Download PDF

**Files in this Data Supplement:**

- Adobe PDF - aat8520\_SM.pdf
