# Supplementary material for: The architectural design of smart ventilation and drainage systems in termite nests
Source: Sci Adv. 2019 Mar 22;5(3):eaat8520. doi: 10.1126/sciadv.aat8520 (PMC6430624; doi:10.1126/sciadv.aat8520)
Supplement: Download PDF [file aat8520_SM.pdf]

Science Advances
AAAS

advances.sciencemag.org/cgi/content/full/5/3/eaat8520/DC1

# Supplementary Materials for

## The architectural design of smart ventilation and drainage systems in termite nests

Kamaljit Singh*, Bagus P. Muljadi, Ali Q. Raeini, Christian Jost, Veerle Vandeginste, Martin J. Blunt, Guy Theraulaz, Pierre Degond

*Corresponding author. Email: kamaljit.singh@imperial.ac.uk

Published 22 March 2019, *Sci. Adv.* **5**, eaat8520 (2019)
DOI: 10.1126/sciadv.aat8520

**This PDF file includes:**

Air Percolation Analysis
Fig. S1. XRD analysis of the nest material.
Fig. S2. Subset selection for the smaller pores in the Senegal nest.
Fig. S3. High-resolution x-ray microtomographic images.
Fig. S4. REV analysis.
Fig. S5. Four-phase separation of the Guinea nest sample.
Fig. S6. Computation of percolation threshold.
Fig. S7. Air percolation analysis in the outer wall of the termite nests.
Table S1. Mineral compositions of the Senegal and Guinea nest material obtained from the XRD analysis.
Reference (*46*)

## Air Percolation Analysis

We computed a percolating path (see the Materials and Methods section) of maximum inscribed spheres (fig. S7A and fig. S7B) extending across the porous sample (fig. S7C). From all the inscribed spheres in the pore space (fig. S7C), we estimate the radius of the largest sphere that fits into the pore space and allows the connectivity (percolation) of spheres across the sample. This radius, called the threshold radius, is used to estimate the threshold capillary entry pressure required for air invasion into the water-saturated sample using the Young-Laplace equation, $P_c = 2\sigma k$ , where $P_c$ is the capillary pressure, $\sigma$ is the surface tension between water and air = 72.75 mN/m at 20°C (*46*). The mean curvature of the water-air interface $k$ was calculated from the threshold radius of the inscribed sphere of the percolating cluster $(r)$ using $k = \cos\theta/r$, where $\theta$ is the contact angle at the three-phase contact points of grains, water and air. A contact angle of 20° is used in the analysis (*36*), which represents strongly water-wet (hydrophilic) conditions. For the particular case of the wall of the Senegal nest, we found a threshold radius of 60 µm for percolation of the maximum inscribed spheres across the sample. This threshold radius covers all the larger pores (compare the pore size distributions of percolating pores in fig. S7D and the pore size distribution of the nest in Fig. 3G). On the other hand, the Guinea nest samples show smaller threshold radii 25-32.5 µm. These values are consistent with the pore size distribution shown in Fig. 3G.

The smaller pores in the subsets of the outer wall of the Senegal nest have a significantly lower threshold in the range 7.5-10 µm. The pore size distribution of the percolating cluster of the smaller pores, as expected, is towards the left side of the distributions of both nest samples (fig. S7D). The Senegal random pack shows a threshold value of 15 µm, which is close to that for the smaller pores of the Senegal nest. This is consistent with the overall pore size distribution of the smaller pores and the pores of the Senegal random pack (red and yellow curves in Fig. 3G).

The calculated capillary entry pressure required for air to percolate through the samples of Senegal and Guinea nest wall is in the range of 2.28 to 5.47 kPa corresponding to a water column height of 0.23 to 0.56 m respectively. This is significantly lower than that of the smaller pores of the subsets of the wall of the Senegal nest sample, which is in the range of 13.67 to 18.23 kPa, corresponding to a water-column height of 1.39 to 1.86 m.

These findings illustrate that if the rain water fills all the pores of the nest wall, the water in the larger pores can still percolate into the ground soil along with the infiltrating water-air front (once the rain stops), driven by a combination of gravity and the capillary forces. The higher permeability of the larger pores allow water to drain at a higher rate. The drainage of water will help to re-establish ventilation and $CO_2$ exchange through the larger pores of the outer wall of the nest.

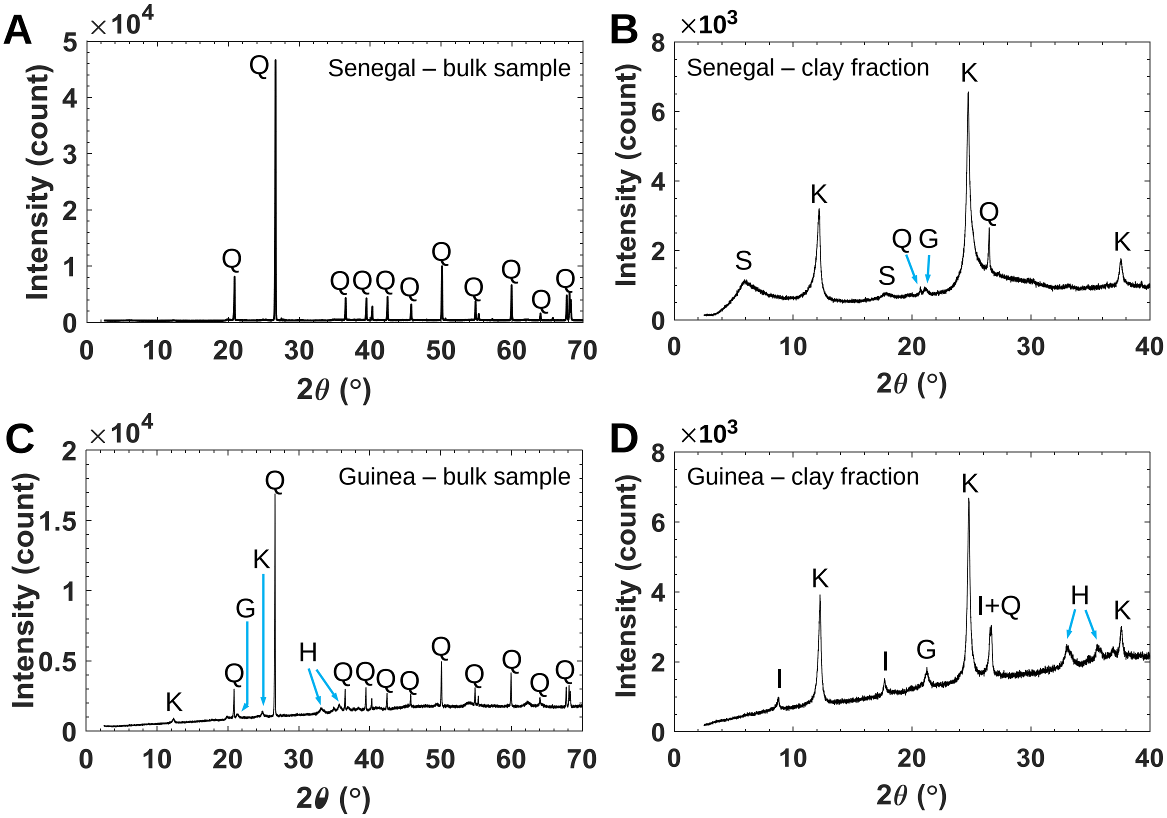


**Fig. S1. XRD analysis of the nest material. A**, **B**. X-ray diffraction data of the bulk Senegal sample (A) and isolated clay fraction (B). The majority of the Senegal nest material consists of quartz minerals with smaller fractions of clay (table S1). **C**, **D**. X-ray diffraction data of the bulk Guinea sample (C) and isolated clay fraction (D). The Guinea sample consists of large fraction of clay and metallic elements. Here, Q represents quartz, S – smectite, K – kaolinite, G – Goethite, H – Hematite, and I – illite.

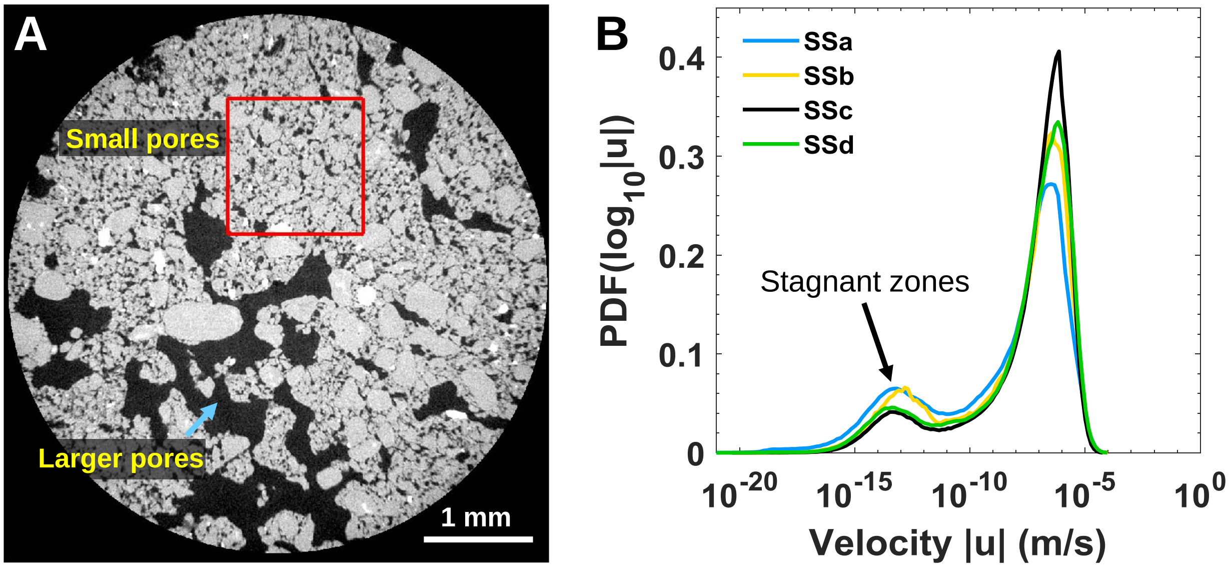


**Fig. S2. Subset selection for the smaller pores in the Senegal nest. A**. To compare the flow behavior and morphological differences, various subsets (SSa to SSd) were taken by isolating the smaller pores of the Senegal nest. The red box shows the location of one of the subsets (SSa with size 250×250×250 pixels in x, y and z directions respectively) in the densely-packed part of the nest. The subset SSb was taken from the subset SSa, with a size 150×150×150, to investigate the effect of the size of the cubical subset on flow properties. The size of subset SSc and SSd is 250×250×250 and 230×230×230 respectively, which were taken from different samples. **B**. Navier-Stokes flow simulations were performed on these subsets (SSa to SSd) containing smaller pores only. The plot shows the PDFs of the logarithm of the velocity fields.

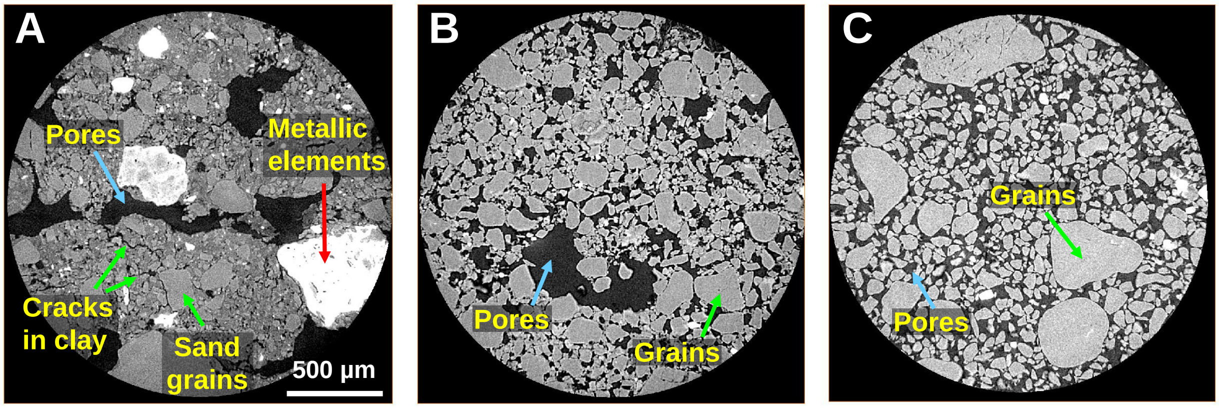


**Fig. S3. High-resolution x-ray microtomographic images. A**. Two-dimensional gray-scale cross-sections of the high-resolution X-ray micro-tomographic images, with a voxel size of 2 µm, of the outer wall of the Guinea nest (A), Senegal nest (**B**) and the Senegal random pack (**C**). The high resolution imaging helps to access the finer details of micro-scale cracks in the clayey parts of the Guinea nest (A). These cracks could occur due to shrinkage during drying of the clayey material.

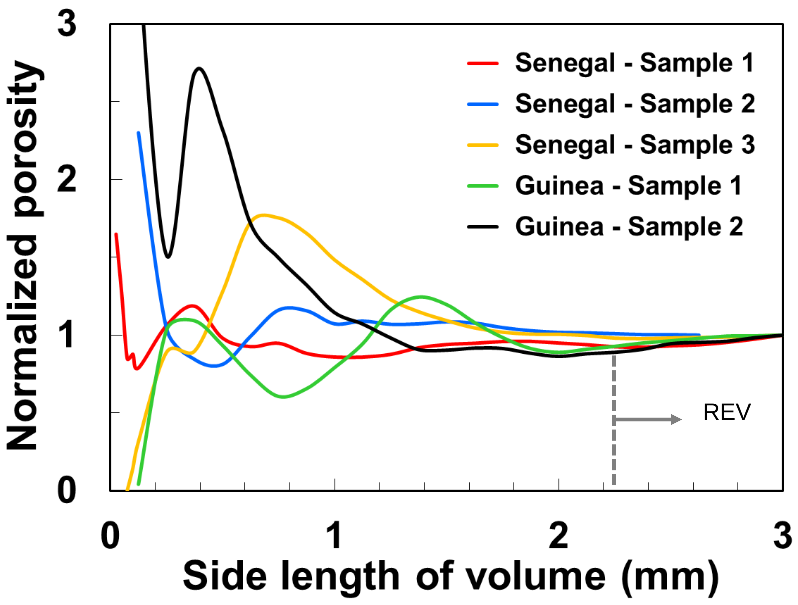


**Fig. S4. REV analysis.** To investigate the effect of image size on the porosity of various nest samples, a representative elementary volume (REV) analysis was conducted, in which the porosity of the cubical subsets with different sizes was computed in each sample. The plot shows the porosity variation as a function of the size of the cubical subset. The porosity is normalized to its final value at 3 mm side length. From these plots, it is clear that a constant value is approached at approximately 2.2 mm.

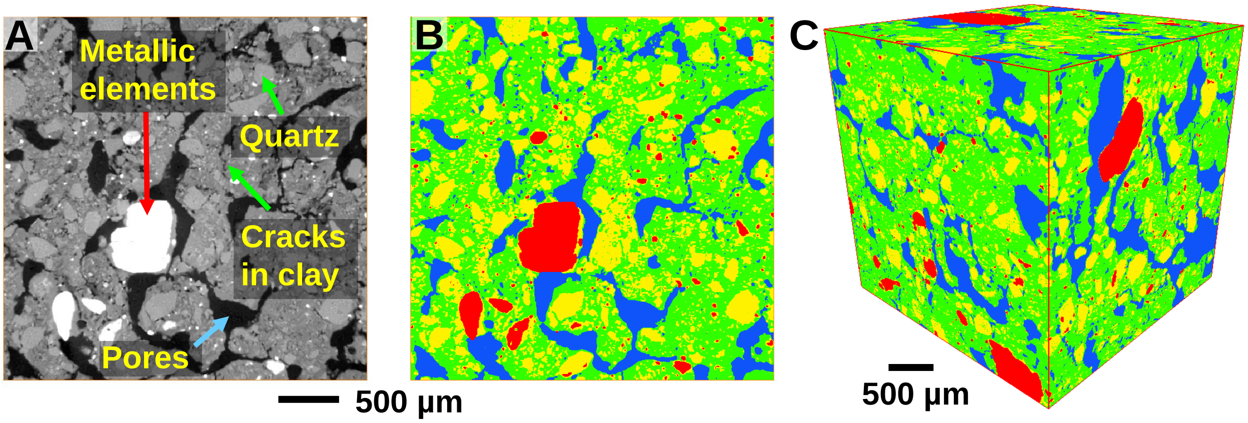


**Fig. S5. Four-phase separation of the Guinea nest sample. A**. Two-dimensional gray-scale cross-section of the X-ray micro-tomographic image showing various components in the Guinea nest sample. **B**. These components were segmented into different phases. Here, quartz, clay, pores and metallic elements are represented by yellow, green, blue and red respectively. The segmented image was used for thermal conductivity simulations in which these phases were assigned their respective thermal conductivity values. **C.** Three-dimensional visualization of the segmented image. The three-dimensional raw gray-scale image is shown in Fig. 3E.

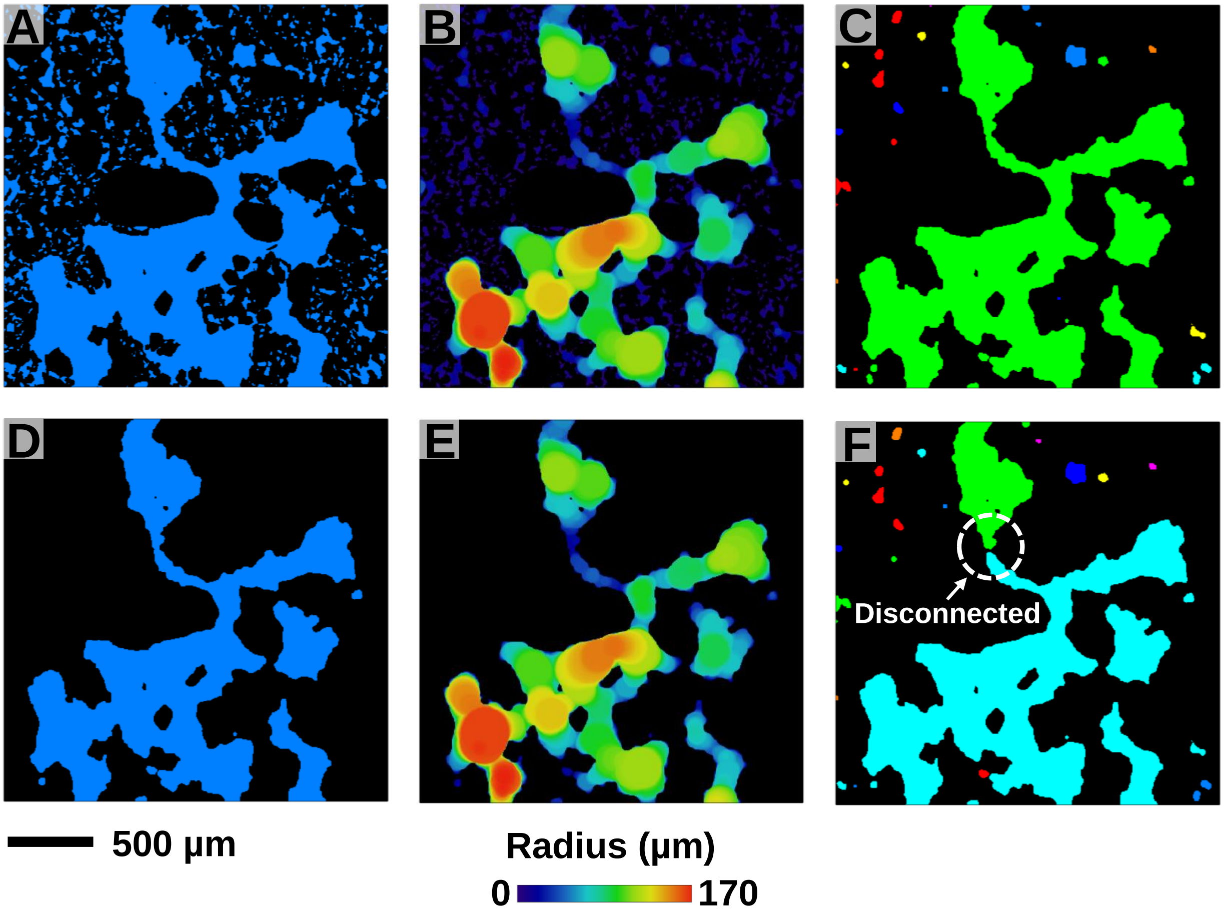


**Fig. S6. Computation of percolation threshold. A**. A two-dimensional cross-section of a segmented image showing pores (blue) and grains (black) in a Senegal sample. **B**. The local thickness map of the pore space representing the radius of the largest spheres that fit into the pore space. **C**. Pore space obtained from thickness map with a threshold radius of 17 µm. The image shows voxels inside spheres with a radius of more than 17 µm. Different colors in the image show disconnected clusters of the pore space. **D**. Isolated connected cluster of the pore space. **E**. The connected cluster obtained from (D) was applied as a mask on image (B) to obtain the thickness map of the connected cluster. **F**. Pore space obtained from thickness map with a threshold radius of 17.5 µm. Different colors in the image show disconnected clusters of the pore space. The dotted circle indicates the location where the connected pore space becomes disconnected and does not span across the image.

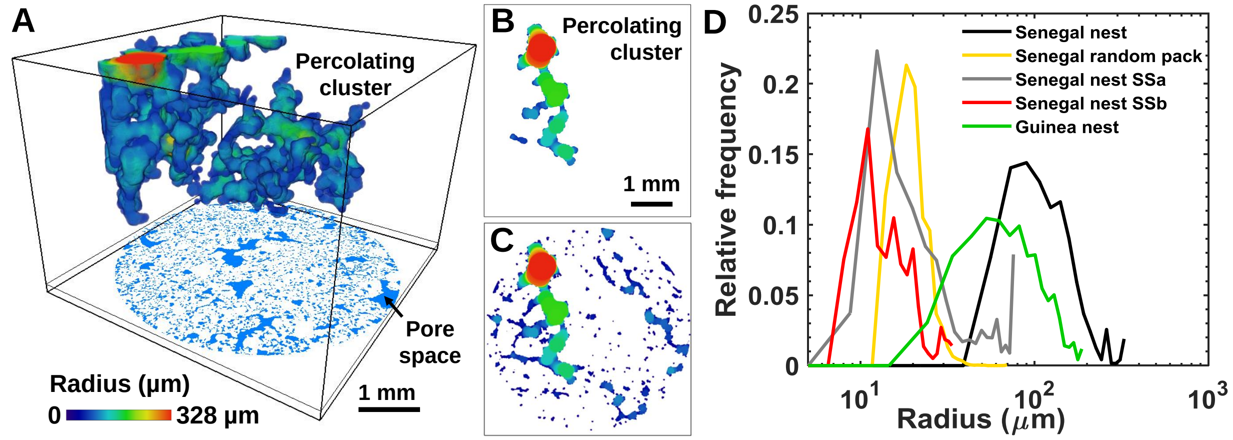


**Fig. S7. Air percolation analysis in the outer wall of the termite nests. A**. Three-dimensional visualization of the air cluster percolating through pore spaces of the outer wall of the Senegal nest (sample 1). The colors in the image show the radius distribution map of the maximum inscribed spheres. Here, the percolating cluster represents a cluster that spans from top to bottom of the image. **B**, **C**. Two-dimensional cross-section of the radius map of the percolating cluster (B) and the complete pore space (C). D. Pore radius distribution of the percolating cluster in the Senegal nest, subsets of smaller pores in the Senegal nest (SSa and SSb), Senegal random pack and the Guinea nest. The subset SSb was taken from the subset SSa, to investigate the effect of the size of the cubical subset on percolation analysis (refer to fig. S2 for further details).

**Table S1. Mineral compositions of the Senegal and Guinea nest material obtained from the XRD analysis.**

| Minerals | Senegal nest (%) | Guinea nest (%) |
|---|---|---|
| Quartz | 87 | 64 |
| Kaolinite | 11 | 18 |
| Smectite | 2 | - |
| Illite | - | 1 |
| Hematite | - | 13 |
| Goethite | - | 4 |
